# Sex differences in endogenous cortical network activity: spontaneously recurring Up/Down states

**DOI:** 10.1186/s13293-017-0143-9

**Published:** 2017-06-15

**Authors:** Charalambos Sigalas, Eleni Konsolaki, Irini Skaliora

**Affiliations:** 1 Neurophysiology Laboratory, Centre for Basic Research, Biomedical Research Foundation of the Academy of Athens, 4 Soranou Efessiou Street, Athens, 115 27 Greece; 2 Psychology Department, Deree - The American College of Greece, Athens, 153 42 Greece

**Keywords:** Sex dimorphism, Neuronal network activity, Development, Aging, Brain slices

## Abstract

**Background:**

Several molecular and cellular processes in the vertebrate brain exhibit differences between males and females, leading to sexual dimorphism in the formation of neural circuits and brain organization. While studies on large-scale brain networks provide ample evidence for both structural and functional sex differences, smaller-scale local networks have remained largely unexplored. In the current study, we investigate sexual dimorphism in cortical dynamics by means of spontaneous Up/Down states, a type of network activity that is exhibited during slow-wave sleep, quiet wakefulness, and anesthesia and is thought to represent the default activity of the cortex.

**Methods:**

Up state activity was monitored by local field potential recordings in coronal brain slices of male and female mice across three ages with distinct secretion profiles of sex hormones: (i) pre-puberty (17–21 days old), (ii) 3–9 adult (months old), and (iii) old (19–24 months old).

**Results:**

Female mice of all ages exhibited longer and more frequent Up states compared to aged-matched male mice. Power spectrum analysis revealed sex differences in the relative power of Up state events, with female mice showing reduced power in the delta range (1–4 Hz) and increased power in the theta range (4–8 Hz) compared to male mice. No sex differences were found in the characteristics of Up state peak voltage and latency.

**Conclusions:**

The present study revealed for the first time sex differences in intracortical network activity, using an ex vivo paradigm of spontaneously occurring Up/Down states. We report significant sex differences in Up state properties that are already present in pre-puberty animals and are maintained through adulthood and old age.

**Electronic supplementary material:**

The online version of this article (doi:10.1186/s13293-017-0143-9) contains supplementary material, which is available to authorized users.

## Background

There are multiple differences in the structure and function of male and female brains of vertebrate species, including humans. At the molecular and cellular level, sexual dimorphism has been observed in several neural processes such as neurogenesis, cell growth, migration, synapse formation, receptor expression, and apoptosis [1, 2]. As a consequence, the formation of neural circuits in the brain, whether structural or functional, is also affected, leading to sex differences in synaptic plasticity [3] and brain organization [4, 5]. Neuroimaging studies have revealed sexual dimorphism in anatomical networks of several brain regions, including both cortical and subcortical regions, such as the volume of white and gray matter [6–9]. At the functional level, functional magnetic resonance imaging (fMRI) and positron emission tomography (PET) studies in animals and humans have demonstrated sex differences in the activation pattern of large-scale neuronal circuits during cognitive tasks (related to emotional perception and memory, fear conditioning and visuospatial properties) or during resting state conditions [10–15]. Sex differences have also been reported in the developmental trajectory of the anatomical and functional neuronal networks of the brain [16]. Moreover, the prevalence of certain neurological diseases, such as dementia, depression, and epilepsy, as well as the response to therapies is also sex-dependent [17, 18].

Surprisingly, most of the studies investigating the sexual dimorphism of functional neuronal networks of the brain have focused only on large-scale networks. In contrast, studies that examine smaller scale local networks are either performed on male subjects exclusively [19–23], or combine males and females, without reporting if sex effects have been accounted for [24–33]. This is an important shortcoming, as it is increasingly acknowledged that an understanding of microscale local network function is essential in order to evaluate the findings at the macroscale network [34]. For this reason, we employed an ex vivo preparation of network activity in the form of spontaneously recurring Up states to examine sex differences in the function of cortical microcircuits. This type of synchronized activity, a characteristic of quiescent brain states, such as slow-wave sleep, anesthesia, and quiet wakefulness, is generated by the intrinsic properties of the cortical networks and is also present in brain slices [35, 36]. As such, it is considered the “default” activity of the cerebral cortex, reflecting endogenous connectivity [35–37]. Up states have been shown to influence information processing in sensory perception [23, 38], while mice with cognitive impairments present alterations in Up/Down state activity [19, 39]. Importantly, using this ex vivo preparation, we overcome the complexity of large-scale networks that can often hinder the discovery of specific contributors to the network mechanism, as we have already shown regarding the nicotinic modulation of Up states [19]. In the present study, we investigate the possible differences in the properties of Up states between male and female mice. We use animals across different stages of their reproductive life––pre-puberty, adult, and old mice––to examine the developmental trajectory between the two sexes. To our knowledge, this the first study that investigates the sexual dimorphism in intrinsic cortical network function along the mouse lifespan.

## Methods

### Animals

C57Bl/6J mice were bred in the animal facility of the Centre for Experimental Surgery of the Biomedical Research Foundation of the Academy of Athens. The facility is registered as a breeding and experimental facility according to the Presidential Decree of the Greek Democracy 160/91, which harmonizes the Greek national legislation with the European Council Directive 86/609/EEC on the protection of animals used for experimental and other scientific purposes. Mice were weaned at postnatal day 21 (P21), housed in groups of 5–9, in 267 × 483 × 203 mm cages supplied with bedding material and kept at a 12–12 dark-light schedule. Food was provided ad libitum. The estrous cycle of female mice was not monitored.

### Brain slice preparation

Coronal brain slices (400 μm) from the primary somatosensory (S1) cortex of the mouse whisker system (i.e., the barrel cortex, S1BF; anterior-posterior from Bregma (A/P), 0.58–1.58 mm; medial-lateral (M/L), 2.5–4 mm) were prepared from pre-puberty (17–21 days old), adult (3–9 months old), and aged (19–24 months old) male or female mice. After the animal was sacrificed by cervical dislocation, the brain was removed and placed in an oxygenated (95% O_2_–5% CO_2_) ice-cold dissection solution containing, in millimolar KCl 2.14, NaH_2_PO_4_.H_2_O 1.47, NaHCO_3_ 27.0, MgSO_4_ 2.2, D-glucose 10.0, sucrose 200, and CaCl_2_.2H_2_O 2.0, measured osmolarity (mean ± SD) 298 ± 5 mOsm, pH: 7.4. Osmolarity was measured using an Osmometer 800 cl (Slamed). Slices were cut using a vibratome (VT 1000S, Leica), placed in a holding chamber with artificial cerebrospinal fluid (aCSF) containing, in millimolar NaCl 126, KCl 3.53, NaH_2_PO_4_.H_2_O 1.25, NaHCO_3_ 26.0, MgSO_4_ 1.0, D-Glucose10.0, and CaCl_2_.2H_2_O 2.0 (osmolarity (mean ± SD) 317 ± 4 mOsm, pH: 7.4) and left to recover at room temperature (RT, 24–26 °C) for at least 1 h before use.

### In vitro electrophysiology

Slices were transferred to a submerged chamber (Luigs and Neumann), where they were constantly perfused at high flow rates (10–15 ml/min) to ensure optimal oxygenation of the cortical tissue [40, 41]. Recordings were performed in “in vivo like” aCSF (composition as above but with 1 mM CaCl_2_) since this ionic solution is thought to better mimic cerebrospinal fluid in vivo [42, 43]. Recordings in each brain slice lasted for 20–40 min (average 28.1 min) and were performed at RT, after at least 30 min incubation in 1 mM (CaCl_2_) aCSF. At temperatures over 30 °C, the metabolic demands of the slice increase, requiring very high aCSF flow rates which impose technical difficulties for successful recordings. Unpublished observations from our lab show that all reported parameters of Up state activity change in a similar way across groups of different ages or sex. Therefore, our recordings were performed at room temperature to increase the yield of successful recordings and reduce the number of sacrificed animals. Spontaneous network activity was assessed by means of local field potential (LFP) recordings (sampled at 5 or 10 kHz, band-passed filtered at 1–3000 Hz)––obtained from cortical layers 2/3 (electrodes were always placed in the middle of layers 2/3) using low impedance (~0.5 MΩ) glass pipettes filled with aCSF. As shown in our previous analysis using simultaneous LFP and intracellular recordings under identical methodology, this type of LFP events correspond to intracellularly defined Up states [19, 20]. Signals were acquired and amplified (MultiClamp 700B, Axon Instruments), digitized (Instrutech, ITC-18), and viewed on-line with appropriate software (Axograph X, version 1.3.5).

### Data analysis

For visualization and analysis of spontaneous LFP events, traces were exported to MATLAB format. The analysis of each recorded trace was performed with MATLAB scripts that automatically detected the deflections in the LFP trace. The data was first low-pass filtered at 200 Hz (3rd order Butterworth filter), and the DC offset was subtracted. Detection of individual LFP bursts was performed with the following automated method: (a) the signal was transformed using the Hilbert transform in order to estimate its envelope [44], and (b) a threshold was applied so as to detect signal segments with fluctuation values larger than 40% of the standard deviation of the entire signal. This threshold was calculated for each trace (data-driven threshold) in order to ensure that the detection procedure is adjusted to the corresponding signal-to-noise ratio of each recording and to the specific properties of each time series (e.g., size and frequency of events). Subsequently, the automatically detected LFP events were visually inspected in order to reject artifacts caused by electrical and/or mechanical noise. Up state duration was calculated as the time interval between the onset and offset of individual events, while Up state occurrence was defined as the number of events divided by the duration of the recording session. The power spectrum of each Up state event was calculated using Fourier transform coefficients and is presented in the conventionally described frequency bands: delta (1–4 Hz), theta (4–8 Hz), alpha (8–12 Hz), beta (12–30 Hz), and gamma (30–100 Hz) range, normalized to the total power of each event in the 1–200 Hz range. Sex and age for each analyzed recording were only revealed after its analysis had been completed.

### Statistics

Statistical comparisons are based on LFP event averages, as in our previous publications [19, 20]. For all parameters, averages were calculated from individual brain slice datasets, as is customary in the analysis of Up states [19, 20, 36, 45, 46]. In summary, a total of 12 slices were recorded in each of the 6 experimental groups. These slices were obtained from 5–7 animals per group, as follows (number of animals/number of slices per animal): pre-puberty male 5/2–3, pre-puberty female 5/2–3, adult-male 6/1–3, adult-female 7/1–3, old-male 6/1–4, and old-female 5/1–4. Since recordings were obtained from one or more slices from each animal, we first tested for the best-fit model, according to the smallest Akaike information criterion, with two fixed effects (independent variables: age and sex) and one random effect (animal identity). For all dependent variables, the best-fit model was the one without random effects, leading to *p* values identical to those of a two-way ANOVA using the same statistical hypothesis testing. We note however, that the reported results are similar even if statistical testing is performed with the animal identity as the random variable. Combined bar graph/scatter plot diagrams for each Up state parameter reported in the manuscript are presented in the Additional file 1.

Data were initially tested for normality and homoscedasticity (Shapiro-Wilk test and Levene’s test, respectively) to explore if the assumptions for analysis of variance (ANOVA) are satisfied. Data regarding the occurrence of Up states were transformed using the Box-Cox transformation [47] to become normally distributed and with equal variance, and parametric tests were performed on the transformed data. The *λ* value for the Box-Cox transform (equal to 0.1533) was defined using an extended dataset beyond the dataset of the current manuscript. For each dependent variable, we performed two-way ANOVA with sex and age as independent variables. Tukey’s post hoc tests were performed whenever applicable, to determine statistical differences between age groups.

## Results

### Female mice exhibit increased Up state activity

LFP recordings of spontaneous network activity were obtained from the primary somatosensory cortex of male and female mice. We used mice of three age groups: pre-puberty (17–21 days old), adult (3–9 months old), and old (19–24 months old), in order to include periods with distinct profiles of sex hormones [48, 49] and to account for possible age effects, as observed in male mice [20]. We first evaluated Up state duration and occurrence, and our results show significant sex differences in both parameters.

**Fig. 1:**
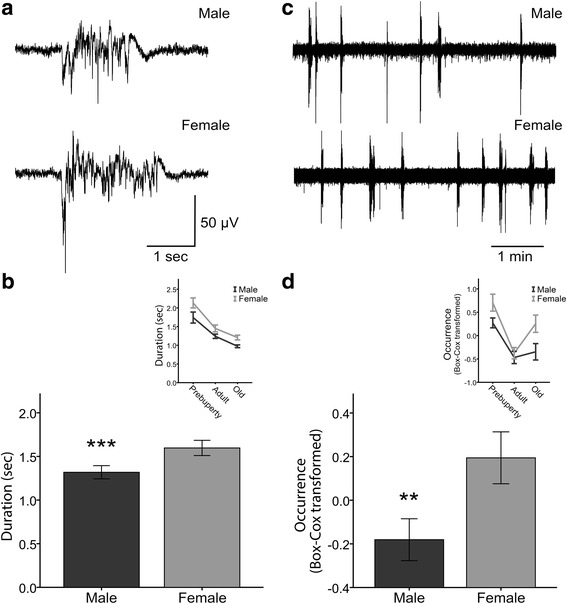
Up state activity is enhanced in female mice. LFP traces at **a** higher and **c** lower temporal resolution obtained from male (*top*) and female (*bottom*) mice. **b** Bar graphs for Up state duration and **d** occurrence (Box–Cox transformed for normality) of male (*black shade*) and female (*gray shade*) mice. Data in *bars* are pooled from all ages tested. *Insets* show line graphs of Up state duration and occurrence in pre-puberty, adult, and old ages. *Graphs* show mean ± SEM. *Asterisks* indicate significant sex differences (two-way ANOVA, ***p < 0.001 and **p < 0.01)

Up state duration in female mice was significantly longer compared to male mice (Fig. 1a, b). Since statistical analysis did not reveal a significant interaction between sex and age (*F*_(2, 66)_ = 0.515, *p* = 0.6, *n* = 12 slices for each of the six groups), data from all three age groups are shown together. There was a significant main effect of sex (*F*_(1, 66)_ = 12.232, *p* = 0.001) and a significant main effect of age (*F*_(2, 66)_ = 39.717, *p* < 0.001), indicating differences among pre-puberty, adult, and old animals. Post hoc analysis showed significant differences among all age groups (Table 1), consistent with our previous findings [19, 20].

**Table 1:** Age-dependent changes in all Up state parameters measured

		Age	
Up state parameter	Pre-puberty	Adult	Old
Duration (sec)	1.94 ± 0.11***###	1.35 ± 0.06§	1.09 ± 0.04
Occurrence (Box-Cox transformed)	0.49 ± 0.11***##	−0.39 ± 0.08	−0.05 ± 0.14
Delta (% relative power)	42.27 ± 1.82*##	49.53 ± 1.96	52.39 ± 1.96
Theta (% relative power)	18.57 ± 0.73	18.55 ± 0.93	17.31 ± 0.90
Alpha (% relative power)	8.92 ± 0.45**###	6.69 ± 0.37	6.22 ± 0.35
Beta (% relative power)	15.09 ± 1.05**##	10.83 ± 0.75	10.35 ± 0.78
Gamma (% relative power)	9.94 ± 0.85	8.63 ± 0.77	9.11 ± 1.05
Positive peak amplitude (μV)	38.7 ± 2.16	32.6 ± 2.72	36 ± 4.37
Negative peak amplitude (μV)	−64.7 ± 3.57*	−46.9 ± 4.39	−57.9 ± 6.56
Positive peak latency (sec)	0.64 ± 0.03***###	0.49 ± 0.02	0.44 ± 0.02
Negative peak latency (sec)	0.62 ± 0.04***###	0.37 ± 0.03	0.27 ± 0.02

Values shown are means ± SEM. Statistically significant differences between pre-puberty/adult, pre-puberty/old, and adult/old mice are indicated by the symbols *, #, and §, respectively

*, #, and §, *p* < 0.05; **, ##, and §§, *p* < 0.01; ***, ###, and §§§, *p* < 0.001

Up state occurrence in male mice deviated from normality (Shapiro-Wilk test, *p* = 0.018) in agreement with previous findings [19, 20]. For this reason, we used the Box-Cox transformation to obtain a normal distribution [19] and performed a two-way ANOVA to estimate the interaction between sex and age. There was no interaction between sex and age (*F*_(2, 66)_ = 1.093, *p* = 0.341, *n* = 12 slices for each group), while there was a significant main effect of sex (*F*_(2, 66)_ = 10.458, *p* = 0.002), indicating that female mice exhibit more frequent Up states compared to male mice (Fig. 1c, d). There was also a significant main effect of age (*F*_(2, 66)_ = 17.221, *p* < 0.001), and post hoc analysis (Table 1) revealed that Up state occurrence was significantly higher in pre-puberty compared to adult and old mice, in line with previous results [19, 20] [back-transformed mean ± SEM (events/min): male pre-puberty, 1.3 ± 0.1; female pre-puberty, 1.95 ± 0.3; male adult, 0.62 ± 0.09; female adult, 0.73 ± 0.07; male old, 0.7 ± 0.1; female old, 1.28 ± 0.2]. Taken together, these results show that while Up state duration and occurrence in the two sex groups follows a similar developmental trajectory, female mice exhibit longer and more frequent Up states compared to male mice.

### Sex differences on Up state power spectrum

**Fig. 2:**
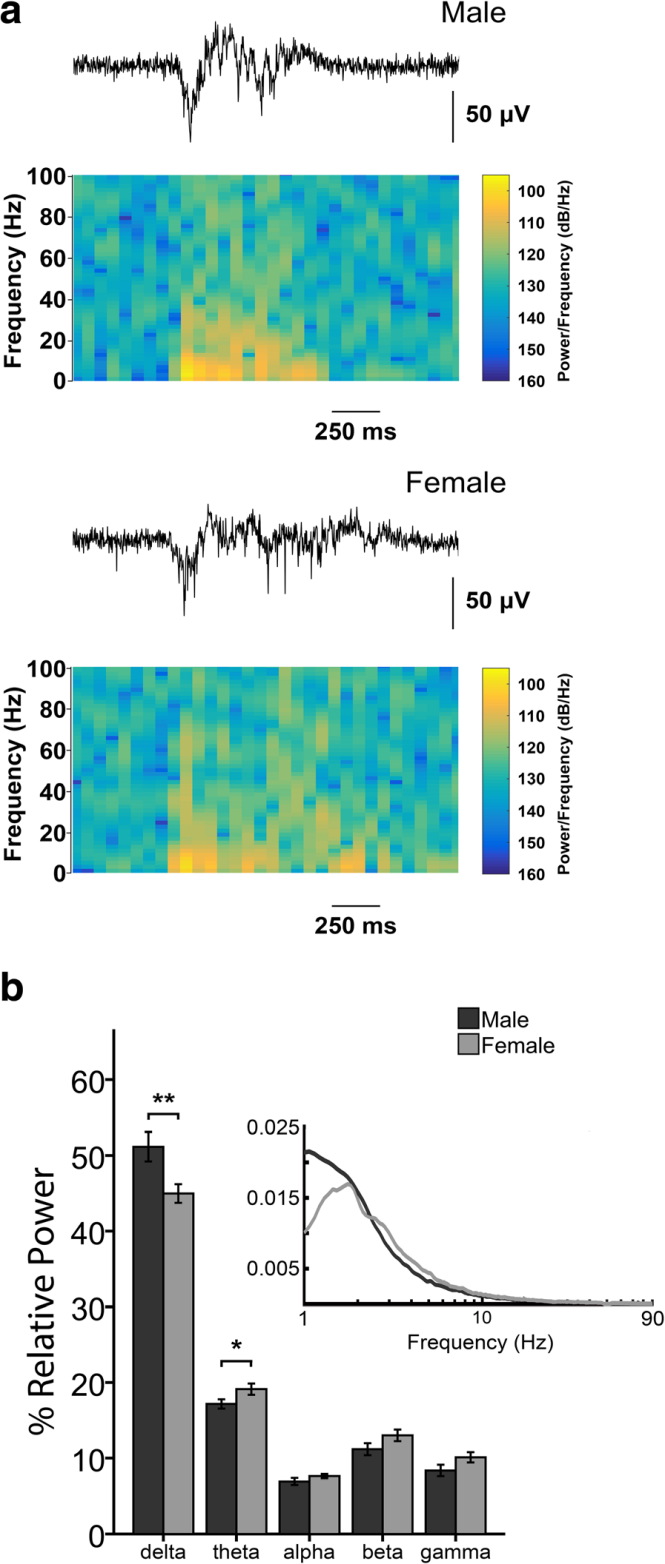
Sex differences in Up state power spectrum. **a** Up state LFP traces and corresponding spectrograms for male (*top*) and female (*bottom*) mice. **b** Bar graphs for Up state relative power percentage in the delta (1–4 Hz), theta (4–8 Hz), alpha (8–12 Hz), beta (12–30 Hz), and gamma (30–100 Hz) range for male (*black*) and female (*gray*) mice. *Inset* shows average relative power percentage without partitioning in the aforementioned frequency bands. Up state relative power percentage represents the normalized power for each band relative to the total power within the 1–200 Hz range for each Up state event. Data in *bars* are pooled from all ages tested. *Graphs* show mean ± SEM. *Asterisks* indicate significant sex differences (two-way ANOVA, **p < 0.01 and *p < 0.05)

We next explored the power spectrum of individual Up state events in male and female mice (power spectrum analysis and autocorrelograms in entire recordings did not reveal rhythmicity in the occurrence of Up states––results not shown). For this analysis, we estimated the power of the conventionally described frequency bands: delta (1–4 Hz), theta (4–8 Hz), alpha (8–12 Hz), beta (12–30 Hz), and gamma (30–100 Hz) ranges. No significant sex differences were found in the absolute power of any of these frequency bands, or in the Up state total power, although there was a trend for lower delta, higher theta, and lower total power in female mice (results not shown). However, given that the variability in Up state amplitude and duration influences the power spectrum, we also calculated the normalized power for each band relative to the total power within the 1–200 Hz range (Fig. 2), which allows for more reliable comparisons within and between recordings [20]. Our results show that Up states of female mice exhibit reduced power in the delta and increased power in the theta range compared to male mice (Fig. 2b). Statistical analysis revealed no interaction between sex and age in any frequency band (*n* = 12 slices for each group): delta (*F*_(2, 66)_ = 0.035, *p* = 0.965), theta (*F*_(2, 66)_ = 0.754, *p* = 0.474), alpha (*F*_(2, 66)_ = 0.286, *p* = 0.752), beta (*F*_(2, 66)_ = 0.986, *p* = 0.378), and gamma (*F*_(2, 66)_ = 0.221, *p* = 0.802). A significant main effect of sex was detected for the delta (*F*_(1, 66)_ = 8.465, *p* = 0.005) and theta (*F*_(1, 66)_ = 4.038, *p* = 0.049) frequency bands, whereas there was no difference in the alpha (*F*_(1, 66)_ = 2.209, *p* = 0.142), beta (*F*_(1, 66)_ = 3.38, *p* = 0.07), or gamma (*F*_(1, 66)_ = 2.848, *p* = 0.096) frequency bands. There was also a significant main effect of age for the delta (*F*_(1, 66)_ = 8.016, *p* = 0.001), alpha (*F*_(1, 66)_ = 13.178, *p* < 0.001), and beta (*F*_(1, 66)_ = 9.245, *p* < 0.001) bands, but not for the theta (*F*_(1, 66)_ = 0.764, *p* = 0.483) or gamma (*F*_(1, 66)_ = 0.547, *p* = 0.581) bands. In all three cases, post hoc analysis revealed that the differences were identified between pre-puberty and adult or old mice, but not between adult and old mice (Table 1). These results reveal sex-dependent alterations in the organization of network oscillations during Up state activity, with female mice exhibiting reduced power in the slowest frequency range (i.e., the delta band) and increased power proportion in the theta band. Despite these differences, the age-dependent changes in the Up state power spectrum are similar in both sexes.

### Sex differences in the profile of Up state peak amplitude

Lastly, we examined the characteristics of the Up state peak amplitude in males and females. For each event, we measured the positive and negative peak voltage, as well as the latency to reach each peak (from Up state onset).

**Fig. 3:**
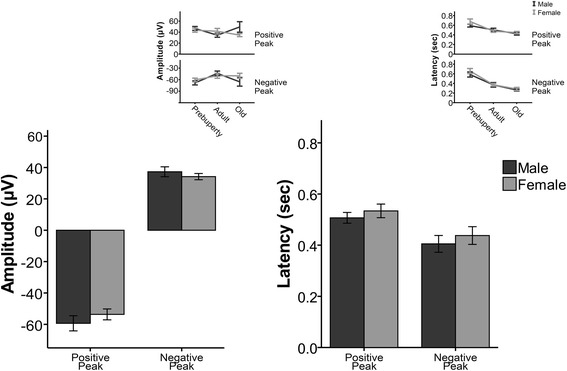
Characteristics of Up state peak amplitudes are similar in both sex groups. Bar graphs for Up state peak amplitude voltage (*left*) and latency (*right*) of male (*black*) and female (*gray*) mice. Data in *bars* are pooled from all ages tested. *Insets* show line graphs of Up state peak amplitude voltage and latency in pre-puberty, adult, and old ages. *Graphs* show mean ± SEM (two-way ANOVA)

We found that the peak amplitude parameters are not different between males and females (Fig. 3), in contrast to the sex differences in the duration, occurrence, and spectral power. There was no significant interaction between sex and age for either peak voltage (positive peak, *F*_(2, 66)_ = 2.216, *p* = 0.117; negative peak, *F*_(2, 66)_ = 1.223, *p* = 0.301; *n* = 12 slices for each group) or their respective latencies (positive peak latency, *F*_(2, 66)_ = 1.382, *p* = 0.258; negative peak latency, *F*_(2, 66)_ = 0.414, *p* = 0.662). Also, the main effect of sex was not significant for either peak amplitude or latency parameters (positive peak amplitude, *F*_(1, 66)_ = 0.718, *p* = 0.4; negative peak amplitude, *F*_(1, 66)_ = 0.979, *p* = 0.326; positive peak latency, *F*_(1, 66)_ = 0.969, *p* = 0.328; negative peak latency, *F*_(1, 66)_ = 0.961, *p* = 0.33).

Nevertheless, there was a significant main effect of age for the negative (*F*_(1, 66)_ = 3.265, *p* = 0.044) but not the positive peak amplitude (*F*_(1, 66)_ = 0.931, *p* = 0.399), as well as both latencies (positive peak latency, *F*_(1, 66)_ = 18.619, *p* < 0.001; negative peak latency, *F*_(1, 66)_ = 39.008, *p* < 0.001). Post hoc analysis (Table 1) revealed that the pre-puberty mice had longer latencies for both positive and negative peak compared to adult and old mice and also larger negative peak amplitude than adult animals. Finally, there was a marginally significant difference between adult and old mice in the negative peak latency (*p* = 0.05). These results reveal that both male and female mice exhibit similar age-dependent changes in the characteristics of Up state peak amplitudes.

## Discussion

The identification of sex differences in brain structure and function is a prerequisite for understanding how male and female brains process information and handle disruptions in neuronal homeostasis. While previous studies have revealed significant sexual dimorphism in the structural elements of the brain [1] as well as in large scale global networks [10–15], the study of the intermediate level of analysis, the cortical microcircuit, has been almost completely neglected. In the current study, we employed an established model of network activity in the form of spontaneously recurring Up states to explore cortical dynamics in male and female mice across the lifespan. This type of synchronized persistent activity, which is generated by the intrinsic local circuitry of the cerebral cortex and reflects the underlying endogenous connectivity, offers an ideal tool with which to explore the manifestation of functional differences at the microcircuit level. We report significant sex differences in Up state properties that are already present in pre-puberty animals and are maintained through adulthood and old age.

### Sources of variability between male and female animals

Differences between male and female animals can be attributed to several distinct, albeit potentially interacting, factors. The secretion profile of gonadal hormones changes during the mouse lifespan, in a manner similar to humans. There is an initial surge during gestation and first postnatal days, followed by suppressed release during the infantile and pre-puberty periods. Subsequently, the release of gonadal hormones gradually rises with the onset of puberty until it reaches adult levels and then declines with aging [48–50]. The effects exerted by these hormones on phenotype and behavior are traditionally classified in two types: (1) “organizational,” which are permanent effects caused by secretion of gonadal hormones during the sensitive periods of development in fetal and neonatal life, and (2) “activational,” which are acute and reversible effects caused by hormonal secretions that start in puberty and continue through adulthood [48, 51]. Recent research, however, suggests that organizational effects might continue during puberty and adolescence [52]. In the current study, we have focused on three age groups––pre-puberty, adult, and old––with different secretion profiles of gonadal hormones, in order to investigate for age-dependent sex differences that could be linked to organizational/activational effects.

We find that spontaneous Up states exhibit sex differences that are already present during pre-puberty, suggesting that these effects are likely due to sex-dependent alterations induced during the prenatal and perinatal life. We also found that the differences remain in adult and old ages, indicating the absence of activational effects on the Up state parameters we have measured. It has to be noted, however, that Up state activity in slices may be devoid of immediate effects induced by acute secretion of sex hormones from the gonads because any circulating hormones could be washed off during the course of the experiment. Nevertheless, using our ex vivo preparation, we can assess the dynamics of the local neuronal network which are generated by the intrinsic properties of the constituent elements of the cerebral cortex. In this context, our results suggest that the sex differences we observed reflect alterations in the intrinsic properties of the cortical network induced by organizational effects of sex hormones or effects mediated by sex chromosomes via nonhormonal mechanisms [53]. Experiments with pharmacological manipulations that influence brain masculinization or feminization, or experiments using the four core genotypes model [53, 54], would be required to clarify the source of the sexual dimorphism we observe.

It has to be noted that, as with all experiments in brain slices, we cannot exclude the possibility that differences in in vitro metabolism (e.g., passive supply of oxygen and nutrients) between males and females may contribute to the sex-dependent differences in Up state activity we observed. However, the fact that no differences were observed in Up state amplitude, a parameter affected by the level of passive supply of oxygen [41, 55], suggests a similar metabolic demand and supply in slices of both sexes.

### Potential mechanisms underlying the differences in spontaneous activity

Our results reveal that significant differences exist at the microcircuit level, as indicated by the greater Up state occurrence and duration values observed in female animals, as well as the different proportions in low-frequency oscillations. Since Up states reflect the spatiotemporal interaction between excitation and inhibition inherent in the recurrent networks of the cerebral cortex [22, 35], factors that affect this interaction are likely to account for the observed differences in occurrence and duration.

For example, studies have shown that a modest reduction in the net inhibition mediated by low doses of GABA_A_R or GABA_B_R antagonists leads to enhanced Up state duration and/or occurrence [19, 27, 36]. Thus, the increased Up state activity in female mice could reflect sex differences in inhibitory signaling. Consistent with this hypothesis, the expression of GABA_A_R α1 and γ2 subunit messenger RNA is lower in the somatosensory cortex of young and adult female mice compared to age-matched male mice [56]. Moreover, female mice have reduced cortical [^3^H]GABA binding for the low-affinity GABA binding sites compared to male mice [57]. Further studies can explore the specific role of GABA in the sexual dimorphism at the microcircuit level.

Alternatively (or in addition), sexual dimorphism in Up state activity can be the result of differential neuromodulation. Although our data were obtained in isolated cortical slices, we have previously shown that the stimulation of nicotinic acetylcholine receptors (nAChRs) by endogenously released ACh reduces Up state duration and occurrence in brain slices of the somatosensory cortex of adult and old male mice [19]. In addition, activation of muscarinic AChRs and noradrenergic receptors abolishes spontaneous and evoked Up states in thalamocortical slices of adult mice [58]. Hence, if either of these systems exhibits sexual dimorphism, it could at least partly account for the observed differences we detect between males and females. In agreement with this scenario, it has been found that the expression of the α4 subunit of the nAChRs in the cerebral cortex was lower in male compared to female rats, although the differences were restricted to early adolescence [59]. In addition, whole cell current responses to nicotinic stimulation in layer VI neurons were larger in male adolescent mice [60]. It is thus possible that slices from male mice exhibit larger nicotinic currents, inducing greater changes in the balance between excitation and inhibition in such a way that Up state duration and occurrence is reduced.

In the current study, we have also observed sex differences in the relative power of delta and theta frequency bands. While previous reports have shown that Up states contain oscillations of variable frequencies [20, 29] that differ between cortical regions [61] and among different ages [20], to our knowledge, this is the first study to document sex differences in the spectral power of Up state activity. Although it is unclear whether the oscillations within Up states relate to cognitive processes, they are thought to reflect characteristics of network organization and its efficiency in generating such rhythms in different brain states [61].

### Similarities in Up state activity between male and female animals

Despite the differences discussed above, we found that several other Up state parameters were indistinguishable between males and females. These included the maximum positive and negative peaks of Up state events as well as the latency to reach them. Up states largely result from the synchronous manifestation of synaptic currents across the recurrent neuronal network [35]. Thus, the LFP amplitude of an Up state reflects the overall size of the active network and the pool of synapses that participate in the synchronized activity. Furthermore, the time delay between Up state onset and maximum amplitude indicates the timing of network recruitment in synchronized activity, which is exhibited during the transition from the Down to the Up state [61]. The lack of significant sex differences in these parameters suggests that the network dynamics that develop during the transition to the Up state are functionally similar in both male and female mice and support the idea that the generation of these self-maintained depolarized states represent a fundamental operation of cortical networks. An alternative interpretation is that subtle sex differences that would only manifest during a specific phase of the cycle may have been missed in our experiments that grouped all females together.

## Conclusions

The present study revealed for the first time sex differences in intrinsic cortical network activity, using an ex vivo paradigm of spontaneously occurring Up/Down states. This type of persistent activity can be sustained in the absence of subcortical or long-range inputs and reflects the intrinsic functional connectivity of the local recurrent networks within the cerebral cortex. The significance of our findings is highlighted by the fact that Up/Down state activity is considered a basic operation of the cortex and that the mechanisms that generate or modulate this type of activity may form the substrate for cognitive functions such as short-term memory, memory consolidation, or modulation of neuronal activity during attention and an experimental model for the flow of activity through cortical circuits [62]. Our findings could thus contribute in the comprehension of the extended literature on sexual dimorphism in the function of large-scale brain networks during cognitive tasks or even resting state conditions (for a review see [15]). Moreover, our results could form the basis for further investigations of the sexual dimorphism in local networks with respect to pathological conditions related to abnormal cortical network function, as has already been documented in macroscale brain networks during epilepsy and depression [17, 18].

## Additional file

Additional file 1: Figure S1. Combined bar graph/scatter plot diagrams for each Up state parameter reported in the manuscript. Data are shown for each age-sex group as depicted in the legend on the bottom right corner. Each circle represents a slice measurement, while the bar graph shows mean ± SEM. Statistically significant differences are not shown in this figure. (TIF 3333 kb)

## Abbreviations

AChRs: Acetylcholine receptors
aCSF: Artificial cerebrospinal fluid
ANOVA: Analysis of variance
DC: Direct current
fMRI: Functional magnetic resonance imaging
GABA: Gamma-aminobutyric acid
GABA_A_R: Gamma-aminobutyric acid receptor alpha
GABA_B_R: Gamma-aminobutyric acid receptor beta
LFP: Local field potential
nAChRs: Nicotinic acetylcholine receptors
P: Postal natal day
PET: Positron emission tomography
RT: Room temperature
S1: Primary somatosensory cortex
S1BF: Barrel cortex
SD: Standard deviation
SEM: Standard error of the mean
